# Exploring the Challenges of Implementing Managers' Competency Assessment Center in the Health System: A Phenomenological Study

**DOI:** 10.4314/ejhs.v34i6.2

**Published:** 2024-11

**Authors:** Maryam Naghshi, Ali Janati, Samira Raoofi, Rahim Khodayari-Zarnaq

**Affiliations:** 1 Department of Health Policy and Management, School of Management and Medical Informatics, Tabriz University of Medical Sciences, Tabriz, Iran; 2 Department of Healthcare Management, Research Center for Evidence-Based Health Management, Maragheh University of Medical Sciences, Maragheh, Iran

**Keywords:** Leadership, assessment, management, health system, selection

## Abstract

**Background:**

The health system needs competent managers to ensure and improve the health of people and manage resources, if managers are chosen correctly. The Managers' Competency Assessment Center is a popular and effective method for selecting, promoting, and developing management competencies. The present study aimed to explain the challenges of implementing an Assessment center in the health sector.

**Methods:**

The present study was conducted with a qualitative and phenomenological approach in 2023 at Tabriz University of Medical Sciences. Interview data were collected through semi-structured interviews with 33 key informants including official assessors of the center, assessed managers by Assessment Centers, senior managers, and executive officers of the center for assessment of the competence of managers. then analyzed by the content analysis method through the software MAXQDA-18.

**Results:**

The results of the present study were categorized into two main themes and nine sub-themes, which are internal challenges, including challenges related to assessed managers, assessors, competency assessment tools, assessment process, and Challenges of training courses for the development of managers and the external challenges including extra_ organizational, organizational, cultural, and political challenges.

**Conclusions:**

The establishment of Assessment Centers within the health system is relatively recent. However, their implementation faces both external and internal challenges. To address this, policymakers should systematically analyze these challenges, prioritize them based on significance and feasibility, and then collaborate with other national centers and draw from international experiences.

## Introduction

Competency assessment is one of the ways to assess managers for their selection, promotion, and development. Competence is a set of individual characteristics that can be considered in four dimensions: the general skills of the individual, the knowledge required to apply the skills, how the individual performs, and the context in which the performance occurs (1). Assessment Center (AC) that assesses the competencies has a higher reliability and validity (2).

The use of competency Assessment Centers has a long history (3). Competency Assessment Center exercises have been around since World War I, and their longevity is a testament to their success (4). Competency Assessment Center is a useful tool for assessing the competence of managerial candidates (5). Assessment techniques are developed or selected to elicit a variety of behaviors and other information related to selected competencies (6). Several assessors are in charge of rating the behaviors of the appraises; simulation exercises with related jobs are used in the Competence Assessment Center (7, 8). Using multiple assessment tools such as group discussion without a leader, psychometric tests, role-playing games, and multiple assessors to assess traits helps triangulate the findings of a trait (9).

Several studies have been conducted on competencies, including the study in India, Technical competencies, personal competencies, customer-oriented competencies, time management competencies, innovation, teamwork, business awareness competencies, and presentation competencies that increase the effectiveness of organizations (10).

The Assessment Center implementation is not easy, and there is a possibility of a dream with challenges and problems in implementation in different contexts (11-13). In Iran, the first Assessment Center was established in industrialized organizations in 2013 (14), and the health system in 2019. The assessment centers of the Ministry of Health, Treatment, and Medical Education, due to newness, need to examine implementation challenges and problems to stabilize their position and increase credibility along with other measures. This study aimed to explain the challenges of implementing the center's approach to measuring competence in Iran's health system.

## Methods

The present qualitative study of a phenomenological type was done by semi-structured interview method at Tabriz University of Medical Sciences and the ethical license number IR.TBZMED.REC.1401.171 of the Ethics Committee of the Research Vice-Chancellor in 2023. Because the qualitative method has been introduced as a specific and distinct method for a deeper understanding of a concept in a specific socio-cultural context, this method was chosen to conduct this study (15, 16).

**Study design and population:** The research community included executive officers of the Assessment Center, assessors, assessed managers, and senior managers of the assessed persons. The inclusion criteria were People who volunteered to participate in the research and agreed to provide their information to the research team and had the following conditions: Executive officers of the Assessment Center, country assessors who have been working in the Assessment Center for at least 6 months, managers who were assessed at operational and middle levels in the center, and senior managers who were directly responsible for the assessed managers. People who did not want to participate in the study were excluded from the study.

To select the participants was used of the purposeful sampling method then the snowball sampling method. Since in qualitative studies, the sample size is not determined in advance, data collection continued until the point of saturation and was determined based on the opinions of the research team. In this study, data reached saturation after conducting 30 semi-structured interviews; to ensure data saturation 3 more interviews were also conducted (Table 1).

**Table 1:** Characteristics of the interviewees

Property		Abundance	Percent
Gender	Man	19	57.57
	Female	14	42.43
Degree of Education	Masters	8	24.24
	Masters	7	21.21
	Ph.D.	18	54.55
Average Work Experience		23	
Average Managerial Experience		8	
Assessor		14	42.42
Assessed Managers	Accepted	4	12.12
	Needs to be developed	2	6.06
	Fielded	1	3.03
Executive Officer of the Center		9	27.27
senior managers		3	9.1

**Data collection method**: To collect data, the common method of qualitative studies was used, i.e. semi-structured interviews using a semi-structured interview guide that included several open questions. This guide was designed based on previous studies and expert opinions (Table 2).

**Table 2:** Interview guide for AC challenges

Interview Questions
What is the Assessment Center?
what are its goals?
What are the challenges of the Assessment Center?
How is the validity of the Assessment Center?
Which tools/exercises in the Assessment Center do you not consider appropriate?

**Semi-structured interview**: Researchers identified participants and invited them to interviews via email. Interview times and locations were arranged based on participants' preferences. Informed consent was obtained orally, allowing participants to be recorded and leave the study at any time. For those who declined voice recording, researchers took notes during the interviews. After 33 interviews, data saturation was achieved, with interview durations ranging from 60 to 70 minutes.

**Data analysis method**: After each interview and meeting, we analyzed the data from semi-structured interviews using MAXQDA-2018 software. MAXQDA software program is useful in reading full texts, correct data management, complete content analysis and complete communication of concepts, the ability to use coding techniques to review and classify texts methodically, and the possibility of simultaneous analysis of several used interviews (17).

After each interview, the interviewer implemented the interviews in the Word text. the research team members reviewed, approved, and entered the interview texts into the MAXQDA 18 software for analysis.

The first researcher from our team coded the content of each interview. to ensure reliability, the second and third researchers approved the codes. We read through the text multiple times to understand the text and then extracted the relevant codes line by line. We assigned the same codes to similar concepts, and after coding all the texts, we categorized the final codes into sub-themes and then into main themes using software. We extracted a table of the themes and discussed and finalized the analysis.

we used a conventional content analysis method to analyze the interview data:

- Reading the text multiple times to understand it
- Coding (identifying concepts and themes)
- Clustering within topics
- Making a summary table
- Writing and explaining phenomena

We used consistency in study methodology, data analysis, and data collection to increase study consistency. We used purposive and snowball sampling methods to include knowledgeable people in the field. An outsider and a qualitative research expert reviewed and verified data transferability. We sent a summary of the interview results to the participants for approval.

To ensure the stability and strength of qualitative data, we used four criteria verifiability, reliability, transferability, and consistency. We collaborated with members of the research team and an external expert to validate the data. We allocated enough time for the interviews in a quiet place. A semi-structured interview with an interview guide was used to collect data.

## Results

**Figure 1:**
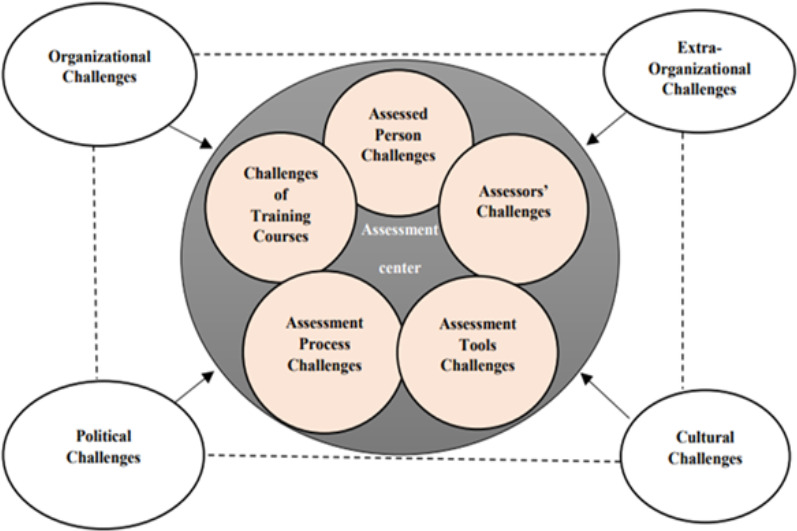
The challenges of the implementation of the competence assessment centers

A total of 33 people participated in the study, and the results were categorized into 2 main themes and 9 sub-themes include internal challenges and external challenges (Figure 1).

Internal challenges are directly affecting the performance of the assessment center. They can occur in the policy-making process, setting policies, issuing executive instructions, and during the implementation phase; this includes all elements and members of the assessment center. It is crucial to correctly execute the entire process, from inviting individuals to the center, conducting the assessment, and finally extracting the results. The current study identifies internal challenges including assessed managers' challenges, assessors' challenges, competency assessment tools challenges, assessment process challenges, and managers' development training course challenges.

External challenges are indirectly affecting the assessment center process and are often rooted in organizational beliefs and culture or political approaches. External challenges include extra-organizational and organizational challenges, cultural, and political (Table 3).

**Table 3:** Main themes, sub-themes, final codes

Main Themes	Sub-Themes	Final codes
**External challenges**	Extra-Organizational Challenges	The inflexibility of the implementation model and framework Lack of organizational chart Improper localization of existing templates Lack of a reference to assess Assessment Centers assess The non-stabilization of the Assessment Center approach Lack of support from policymakers and upstream managers Limited financial resources The risk of increasing administrative bureaucracy with the implementation of the center Not using prominent organizations for assessor training External pressures to appoint people
	Organizational Challenges	Lack of re-assessment of probation managers after development training courses Lack of re-assessment to renew the certificate of competence Introduction of unqualified people as assessors by senior managers Lack of proper assessor training Not setting up the center in all cities Limiting the power of senior managers by assessing managers in the Assessment Center Non-availability of the database of applicants for management positions Designation of the manager outside the assessment process Assessment by the organization itself Uncoordinated actions of some universities Appointment of the manager before the assessment in the AC Lack of structure to assessment in field real job Direction upgrade Uncertain occupation of some occupations Absence definition organizational position assessor of the center Unstable position of managers and premature changes in management The unknown effectiveness of the center in empowering managers The disproportion of the assessment result with the actual competence of the person Limited assessment of managers until appointment
	Cultural Challenges	Belief in the formality of assessment Senior managers' lack of belief in assessment Lack of familiarity of most system managers with management science Ethnic, gender, religious, and political views on the assesses Fear of successor nurturing of senior managers
	Political Challenges	The political nature of some appointments Lack of necessary platform for the cooperation of international organizations in the field of assessment Political instability in the country Failure to assess senior managers
**Internal challenges**	Assessed Person Challenges	The damage caused by the assessors' imitation of the center's process for their subordinates The possibility of adverse influence of some people for the continuation of the activity of the center, whose assessment result was the requirement of competence growth or failure Anxiety caused by assessment in the assessed person Problems of assessing people with mental and moral problems The occurrence of unwanted incidents in the center Non-competitive center for the assessed
	Assessors' Challenges	Inadequacy of the assessor in recognizing examples Possibility Deliberate and inadvertent errors during Assessment Simplification of assessment A limited number of expert appraisers Probability of error in wash-up Differences in the assessment method of assessors The assessor's tendency to give a balanced score to the example Difficulty in choosing an appraiser Problems using the manual for tools by the assessor The difference in the ability level of assessors The inability of assessors outside the organization to assess the field of health and treatment
	Assessment Tools Challenges	Limited to current tools Lack of precise definitions of examples for each competency Lack of an expert team to design measurement tools The difficulty of measuring the validity and reliability of tools discordant in competence scoring in different tools Incompatibility of some tools with the work field of some assessed people Challenges related to examples assessed competencies Tools update problems Non-standard tools The possibility of inefficiency of some tools in recognizing and measuring the mentioned competence
	Assessment Process Challenges	The challenging nature of the assessment process Lack of psychometrics Lack of feedback to the assessor Limited time for assessment Increasing the number of ACs implemented in a short period long implementation process from the introduction of people to the announcement of results Expediency in the center's results Complete lack of coordination in the assessment method Tendency to subjective assessments
		The possibility of differences in taste in the centers of different regions due to the views of the assessors Lack of clarification of the center's process before the assessment The center's inability to measure some competencies Lack of assessment of specialized skills The gap between the actual performance and the individual's performance in the AC
	Challenges of Training Courses for The Development of Managers	lack of Competency development program for managers who need development The effectiveness of development classes is unknown Problems related to conducting face-to-face development classes unit management for organizing AC and conducting competence development courses Theory-oriented development courses Inadequate content of development courses

### External challenges

**Extra-organizational challenges**: The main challenges in this area are a lack of organizational charts and permanent positions for the Assessment Centers, an improper and inflexible adaptation of foreign models, external pressures for appointing people, and inadequate use of suitable organizations for the assessment process.

“The committee for this task is temporary and has no organizational chart. This makes it an unstable mechanism.” (M. 3)

“We copy foreign models without considering our needs and requirements. This destroys many things and makes us unfamiliar with the work.” (M. 10)

**Organizational challenges**: The main challenges in this area are unstable situations and frequent changes of managers, lack of clear and practical guidelines for re-evaluating and renewing the competency of managers.

“The change of managers is a problem; we face obstacles when we start a program and we are in the middle of it.” (M. 14)

“The early management changes of the Ministry of Health are very harmful; the assessment center is very important and it needs stable management.” (M. 19)

**Cultural challenges**: The main challenges in this area are the low familiarity of most managers with management science and art, the fear of senior managers being replaced, and the influence of ethnic, gender, and religious views on the assessment process. These factors make the managers not trust the assessment. The lack of belief in assessment by senior managers, which shows the wrong organizational culture of some organizations, is the most important cultural challenge that more than 80% of the participants mentioned.

“The assessor should only evaluate the general competencies of the person, not his or her personal views. These should be separated.” (M. 23)

**Political challenges**: The main challenges in this area are the political instability of the country and the lack of platforms for the cooperation of international organizations in the assessment field. These conditions also lead to the lack of assessment of senior managers and the appointment of some people based on their political views.

“Our political culture makes some people have connections with people outside the organization and get positions based on their views, and senior managers are not introduced to the organization.” (M. 4)

### Internal challenges

**Challenges related to assess**: The assessment causes anxiety and psychological pressure in the assessed people. There may be a difference between their actual performance and their performance in the center. The assessors imitate the center process for their subordinates.

“There may be a person who is afraid of the tests and cannot show his abilities in the center tests because of anxiety, and he may be eliminated.” (M. 7)

**Challenges related to assessors**: The challenges in this field are the lack of expert assessors, the difference in the assessment method and ability level of the assessors, and the tendency of the assessor to give a balanced score.

People are assessed with mental and moral problems, the possibility of mistakes, the inability of assessors outside the organization to assess the health and treatment field, and simplistic assessment.

“An appraiser may not see behavior or have a different impression and perception of behavior from another assessor, and we will have a difference in the assessment” (M. 17)

**Challenges related to competency assessment tools**: The lack of an expert team for designing assessment tools, the lack of a precise definition of the criteria for determining each competency, and the inconsistency of scoring competencies in different tools have caused the lack of reliability and validity of some assessment tools in different job groups.

“The validity and reliability of the checklists and the obtained results should also be examined.” (M. 8)

**Challenges related to the assessment process**: The limitation of assessment time despite the long implementation process from the time of introducing people to the announcement of results, Increasing the number of ACs implemented in a short period, Tendency to subjective assessments, Lack of clarification of the center's process before the assessment, And Lack of assessment of specialized skills are among the most important challenges.

“Assessment is one day, but the process is long.” (M. 20)

Another challenge is that there is no complete coordination in the evaluation methods, and due to the evaluator's point of view, there is a possibility of differences in taste in the centers of different regions.

**Challenges related to development training courses management skills**: Lack of competency development programs for managers who need development, theory-oriented development courses, unit management for organizing AC, and conducting competence development courses, problems related to conducting face-to-face development classes, and inadequate content of development courses are the most important challenges related to the training and development of managers.

“The development courses should be reviewed and made more operational and the educational content should be updated.” (M. 33)

## Discussion

The current research showed that the Assessment Center is facing 2 major external and internal challenges. Another important external challenge is the influence of cultural factors. The findings of this research are in line with studies (18, 19), to avoid ethnic, gender, religious, and political views on the evaluated people, assessors should have similar context and context culture tools and content. Scenarios should be translated based on valid and high-quality standards, and ACs located in different cultures, in addition to having a suitable structure for such conditions, should have trained assessors familiar with intercultural issues related to it (8, 20).

In line with the present study, in the Lowry PE study, there are challenges such as the cost of the AC, the lack of scenarios, the limited tools, the problems of the tools, the lack of sufficient training of the assessors, and the lack of providing feedback to the assessed managers (21) and in another research unqualified assessors, inappropriate scoring of examples, problems with tools, lack of empowerment of assessors, lack of prior assessment of the AC have been mentioned (22).

In Pattnaik's study, the challenges of the Assessment Centers (ACs) in India included: an inappropriate competency model, inadequate tools, an inappropriate ratio of assessors to participants, insufficient preparation of assessors, variation in ratings by assessors, inadequate feedback sessions, and poor planning (9).

Some of the studies confirmed the validity of the center and raised construct validity problems (23-28). Also, according to the present study, there is a possibility that real results may not be obtained by assessment, which was related to the process of the center and how to select the assessors and empower them in Lowry's study (19, 29). Howard's study results indicate that the challenges of assessment revolve around concerns related to assessment methods, validity, generalizability, participants' reactions, the changing nature of management structure, new applications, and cost-effectiveness. (30) The research indicates that assessment centers demonstrate both general validity and situational specificity (31).

ACs should be by the laws and conditions of the context in which it is implemented, taking into account the context conditions, the standardization of tools concerning competencies is necessary in line with the present study (32, 33). On the other hand, the judgmental nature of the evaluation probably increases the possibility of bias and assessment errors (34).

Evaluation of effectiveness is one of the main pillars of educational needs assessment (35, 36). The ambiguity in the effectiveness of training and development courses and the lack of belief and support of senior managers are the results of the present study, on the basis study of Afsouran et al that will cause serious problems in the empowerment of managers (37). A recent study revealed a significant lack of digital skills among health professional educators. The training programs are necessary to enhance digital competencies to improve the performance of health professional managers and educators (38).

The most important strength of the current research is having a comprehensive look at the ACs including position, structure, the results feedback, and continuous assessment.

In our study, we encountered several limitations. Firstly, certain managers disagreed with our results and were uncooperative. Additionally, some managers lacked faith in the Assessment Center (AC) process, and certain assessors perceived their participation in our research as a threat to their working conditions. Another limitation is that we focused solely on internal and external challenges without examining their interactions. Furthermore, our sample was restricted to a specific Assessment Center, which may impact the generalizability of our findings to other centers. Lastly, in organizations where senior managers lack management skills and oppose succession, competent individuals are not appointed to management roles

In conclusion, this study highlights the direct and indirect impact of internal and external challenges on the performance of Assessment Centers (AC). Internally, challenges relate to evaluated managers, assessors, competency assessment tools, the assessment process, and managerial training and development programs. Externally, organizational and extra-organizational issues, as well as cultural and political factors, play a role.

To enhance AC performance, it is crucial to address both internal and external dimensions. This involves comprehensive attention—from policymaking to implementation. Developing appropriate organizational culture and beliefs, along with making effective decisions to address politically influenced appointments, can significantly affect assessments. Executing the assessment process using professional assessors, reliable tools, and effective training programs contributes to selecting competent managers and developing managerial competencies within the health system. For future research, exploring the interactions between internal and external challenges and using larger samples will enhance the generalizability of findings. Additionally, investigating practical solutions to mitigate these challenges would be valuable.
